# Congenital goiter with areas of signet ring cell differentiation in a juvenile giraffe: a very rare entity

**DOI:** 10.1186/s12917-020-02637-4

**Published:** 2020-10-31

**Authors:** Jinling Wang, Yulin Ding, Jirintai Sulijid, Li Zhao, Shoufeng Lu, Xiaoyu Wang, Yonghong Liu

**Affiliations:** 1 College of Veterinary Medicine, Inner Mongolia Agricultural University, Inner Mongolia 010010 Hohhot, China; 2 Key Laboratory of Clinical Diagnosis and Treatment Technology in Animal Disease, Ministry of Agriculture and Rural Affairs, Inner Mongolia 010010 Hohhot, China; 3 Ordos Zoo Management Institute, Inner Mongolia 017000 Ordos, China

**Keywords:** Giraffe, Thyroid, Congenital goiter, Signet ring cell, Immunohistochemistry

## Abstract

**Background:**

Congenital goiter is a common thyroid metabolic disorder characterized by low levels of thyroid hormone, subsequent secretion of excess thyroid-stimulating hormone (TSH) from the pituitary gland, and compensatory hyperplasia of the glands. The presence of signet ring cells (SRCs) does not provide sufficient evidence for the diagnosis of a thyroid tumor, making histopathological diagnosis challenging. In addition, SRCs can also appear in congenital goiter. Therefore, a comprehensive diagnosis of congenital goiter is warranted based on clinical symptoms, autopsy, histopathology, and laboratory examination.

**Case presentation:**

A juvenile giraffe at the Ordos Zoo in Ordos presented with symptoms of loss of appetite, serious salivation, and slow growth rate since birth. Its height and weight were significantly lower than those of other giraffes of the same age. The animal ultimately died at 17 months of age. Autopsy revelaed that the thyroids were hard, with an uneven surface and with the presence of many small raised follicles, and dense in cross-section. Other organs were visibly atrophic. Histopathologically, diffuse follicles were irregular in size and shape in the hyperplastic goiter. Some follicles were collapsed due to lack of colloids. The follicles were lined by single or multiple layers of hyperplastic follicular cells (HFCs), some of which were exfoliated in the lumen. The HFCs were either cuboidal with eosinophilic cytoplasm and many red small granules or showed SRC differentiation, with nuclei pressed to one edge of the cell and distorted by cytoplasmic mucin that appeared as a single clear vacuole HFCs and as a foamy, multivesicular cytoplasmic material in others. Scattered necrosis of myocardial cells and hepatocytes, cerebral hemorrhage, necrosis of intestinal villi, and obvious atrophy of organs were also observed. Immunohistochemical tests were strongly positive for thyroglobulin and thyroid transcription factor-1 (TTF-1) in the cytoplasm of HFCs.

**Conclusions:**

Here we present a case of congenital goiter with areas of SRC differentiation in the thyroid of a juvenile giraffe.

## Background

Congenital goiter is a common thyroid metabolic disorder characterized by low levels of thyroxine, subsequent secretion of excess thyroid-stimulating hormone (TSH) from the pituitary gland, and compensatory hyperplasia of the glands [1]. It was originally found in humans [2] and was later reported in animals, including Afrikander cattle [3], calves [4], merino sheep [3–5], goats [6], dogs, cats [7], tenterfield terriers [8], and mice [5]. Thyroid hormones affect almost every system of the body; therefore, the possible clinical signs vary. Common signs include growth retardation, developmental abnormalities, disproportionate dwarfism, skeletal abnormalities, impaired mental status, neuromuscular signs, dermatological signs, goiter, dysphagia, and dyspnea [7]. The main morphological features of congenital goiter are the presence of irregular follicles devoid of apparent colloids, elongated columnar follicle epithelial cells, and in many cells, distended rough endoplasmic reticulum vacuoles with free ribosomes [3].

Follicular cells in the thyroid tissue may undergo squamous, oncocytic, or clear cell metaplasia. Of these, the clear cell change with signet ring is the least common variant [9, 10]. The thyroid signet ring cell (SRC) variant refers to the intracytosolic accumulation of thyroglobulin (TG) [11], mucin [11–13], lipids [11, 13], glycogen [11], inclusions [14], lipofuscin, hemosiderin [13], and other substances [15] in thyrocytes. This accumulation leads to the enlargement of the cell body, and the cytoplasm becomes clear with obvious vacuoles. The contents of the vacuoles are colorless or mildly eosinophilic. The nuclei are pushed into a crescent shape, and the whole cell shows a signet-ring appearance [10]. The presence of SRCs in the thyroid may indicate a primary thyroid disease, such as SRC follicular adenoma, papillary thyroid carcinoma, or metastatic SRC carcinoma [16]. Among thyroid tumors, the SRC tumor is more common in adenomas than in carcinomas [17]. Moreover, SRC can also appear in congenital goiter of merino sheep [2]. In this article we report a congenital goiter with areas of signet ring cell differentiation in a juvenile giraffe.

### Case presentation

**Fig. 1:**
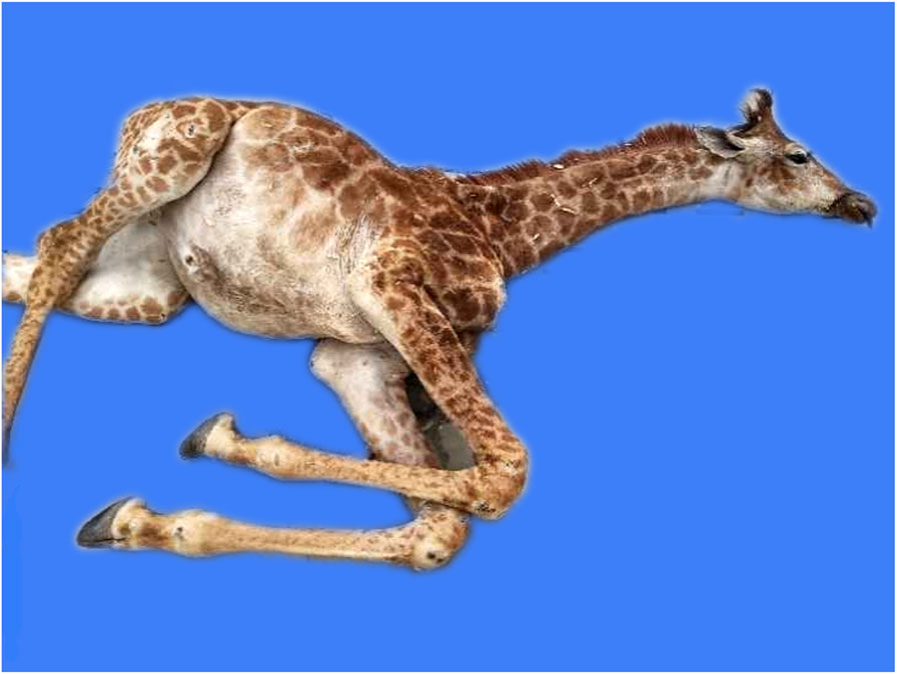
The dead giraffe was emaciated and short for her age

A giraffe named “Lulu” was born with no significant difference in height and weight compared with other giraffes at Ordos Zoo. However, after birth, the giraffe showed poor appetite, salivation, and slow growth. Her daily weight gain was significantly lower than that of other giraffes of the same age. For example, at 8 months old, Lulu was only 255 cm tall, which is typically the height of a 4-month-old giraffe. Lulu experienced extreme weight loss in the 3 months before her death. At 17 months old, Lulu weighed only 144 kg (Fig. 1), which was significantly less than the typical weight of a 1-year-old giraffe (approximately 290 kg).

At 15 months old, Lulu began to show severe salivation, particularly when ruminating, and treatment with Ginseng Jian Pi Wan and stanozolol tablets for 2–3 months was ineffective. Biochemical examination before death revealed significant increases in the levels of globulin, total bile acid (TBA), urea, lactate dehydrogenase (LDH), creatine kinase isoenzyme (CK-MB), α-hydroxybutyrate dehydrogenase (HBDH), amylase (AMY), and alanine aminotransferase (ALT). On the other hand, the levels of calcium (Ca), phosphorus (P), zinc (Zn), albumin (ALB), albumin/globulin (A/G), total bilirubin (TBil), cholinesterase (CHE), prealbumin (PA), glucose (GLU), high-density lipoprotein (HDL-C), carbon dioxide combining power (CO_2_CP), total iron binding capacity (TIBC), and creatinine (CREA) were significantly reduced. Blood tests also revealed significant reductions in monocyte ratio (MONO), eosinophils (EO), eosinophil ratio (EOSR), platelets (PLTs), mean corpuscular volume (MCV), and mean corpuscular hemoglobin (MCH). The red blood cell distribution width (RDW) was significantly increased. On March 22, 2019, Lulu could not stand up on her own after a fall, and she died on March 28, 2019.

**Fig. 2:**
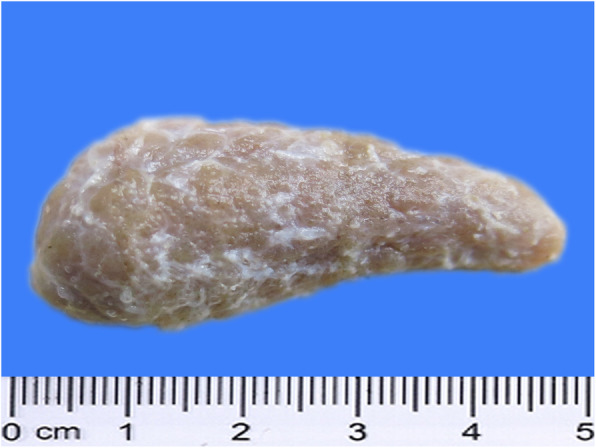
The thyroid gland was hard and the surface was uneven with many small raised follicles (formalin-fixed specimen)

**Fig. 3:**
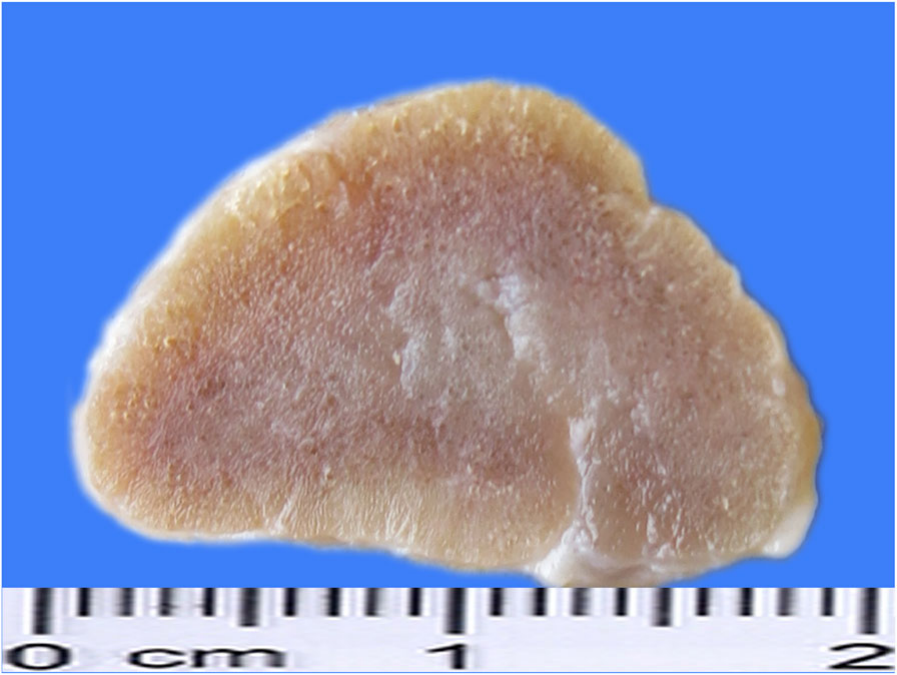
The central area of the thyroid section was hard and grayish-white in color (formalin-fixed specimen)

Autopsy revealed the presence of spindle-shaped thyroid lobes of firm consistency and uneven surface, with grayish-yellow protrusions scattered under the capsule (Fig. 2). The central and peripheral areas of the thyroid section were dense and yellowish-white in color (Fig. 3). No nodular hyperplasia was observed on the surface or section of the thyroid gland. The fat in the sulcus coronarius was visibly atrophic and yellowish and gelatinous in form. The liver was reduced in size and was dark in color. The liver capsule was significantly thickened, with uneven surfaces and sharp edges. The lungs were dense and dark red in color. In addition, the submucosa of the renal pelvis was thickened and gelatinous due to edema. A few small petechiae were visible on the edge of the section of the mesentery lymph nodes and some scattered petechiae were present in the brain section. Colonic mucosal folds appeared dark red in color. Other tissues and organs showed varying degrees of atrophy.

**Fig. 4:**
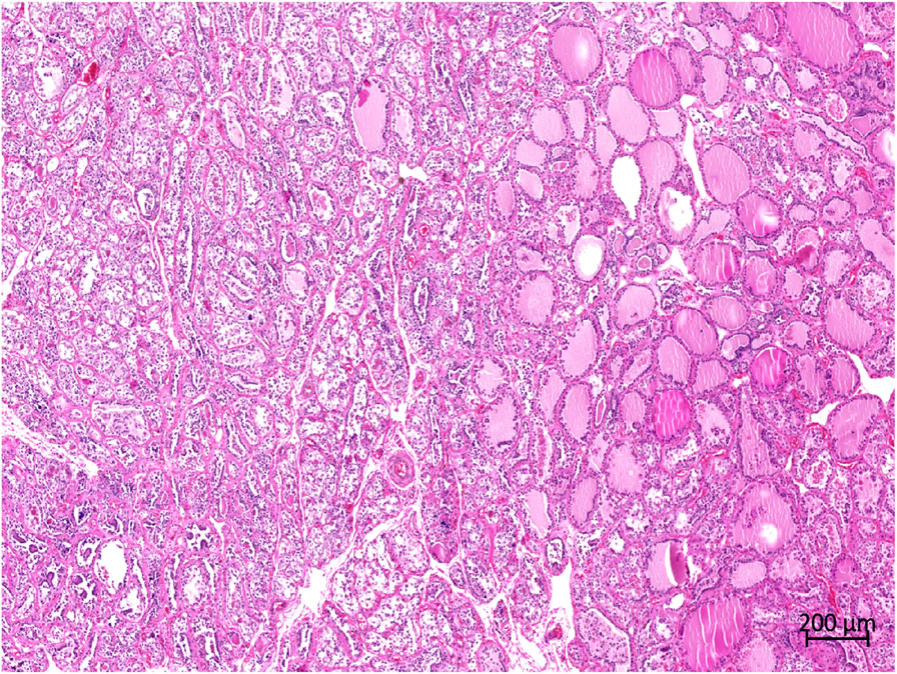
Histopathological features of the thyroid. The diffuse thyroid follicles were irregular, with obvious cell proliferation, disorderly arrangement, and high-cell density. Relatively normal pink thyroid follicles are visible on the right, with pink hormones in the follicular cavity

**Fig. 5:**
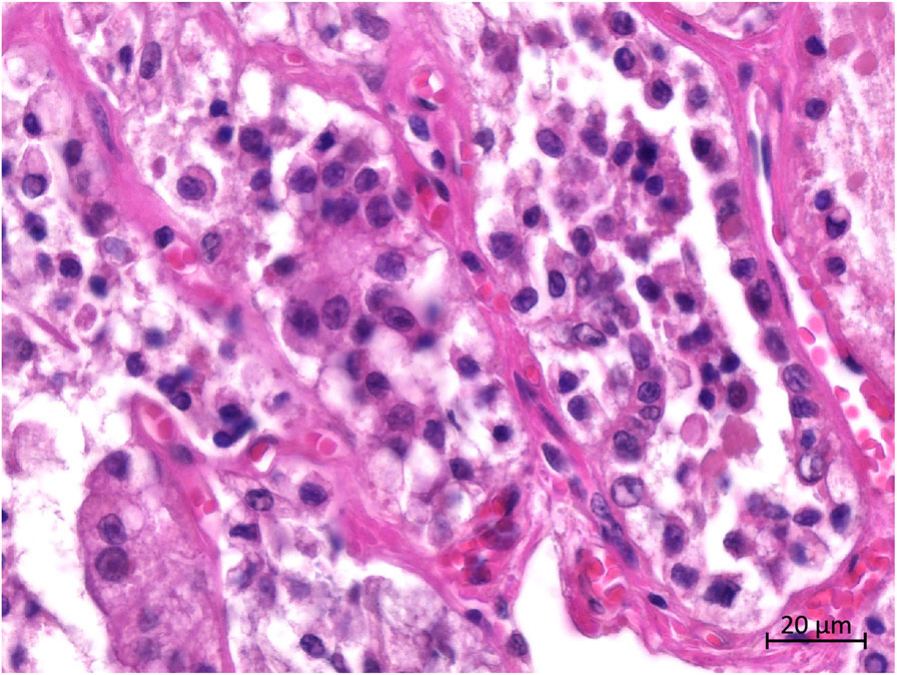
Histopathological features of the thyroid. The follicles were collapsed and lined with single or multiple layers of HFCs

**Fig. 6:**
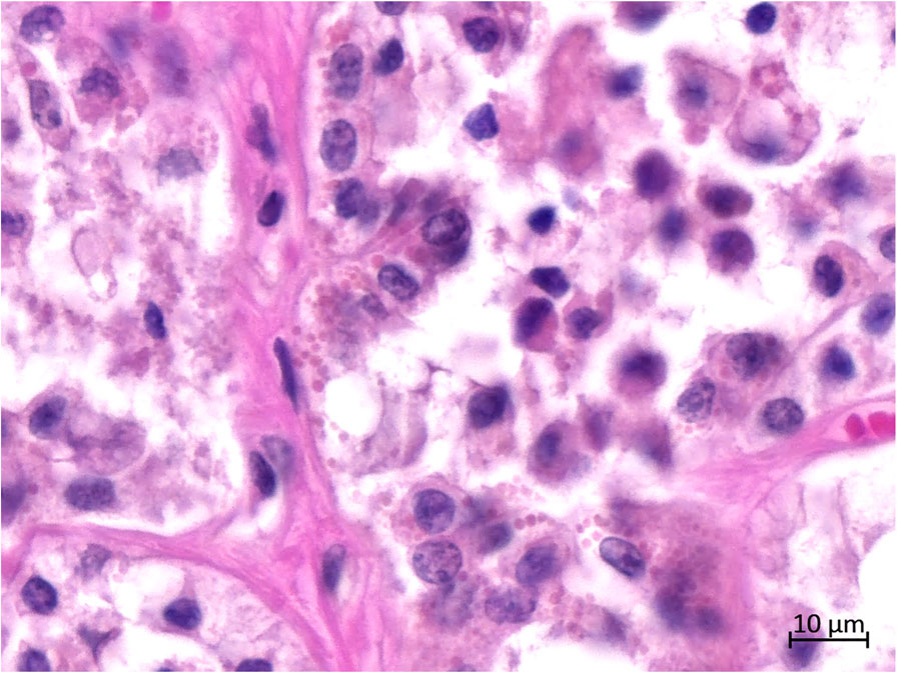
Histopathological features of the thyroid. The cytoplasm of HFCs contained more or less small inclusion-like granules that were red in color

**Fig. 7:**
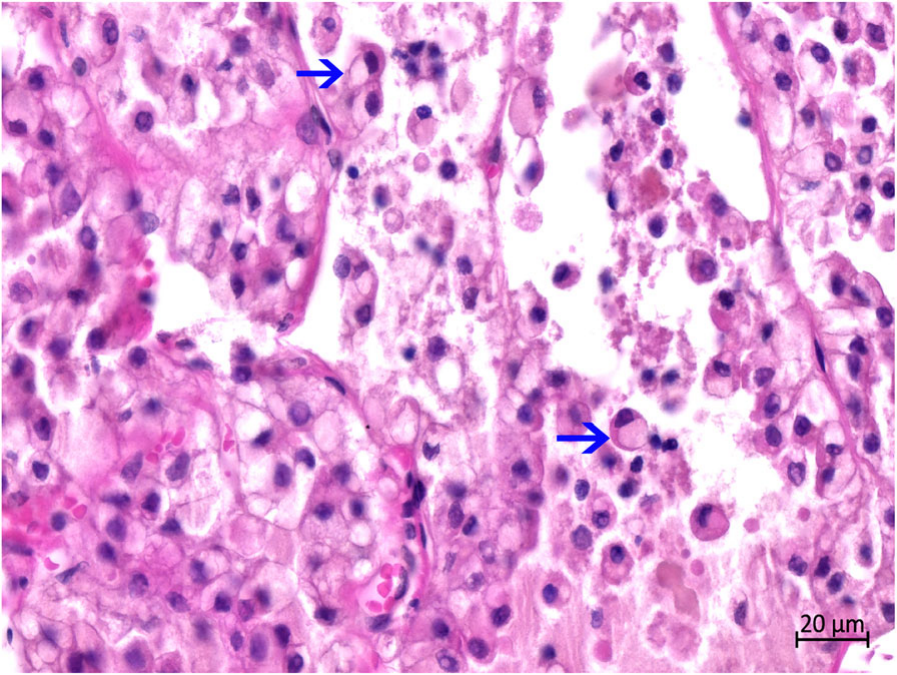
Histopathological features of the thyroid. The HFCs were large in size. Obvious vacuoles and oval eccentric nuclei are visible in the cell cytoplasm. The whole cells showed signet-ring simulation (blue arrow). Numerous signet-ring-like HFCs are lined on the basement membrane of the thyroid follicles (left)

**Fig. 8:**
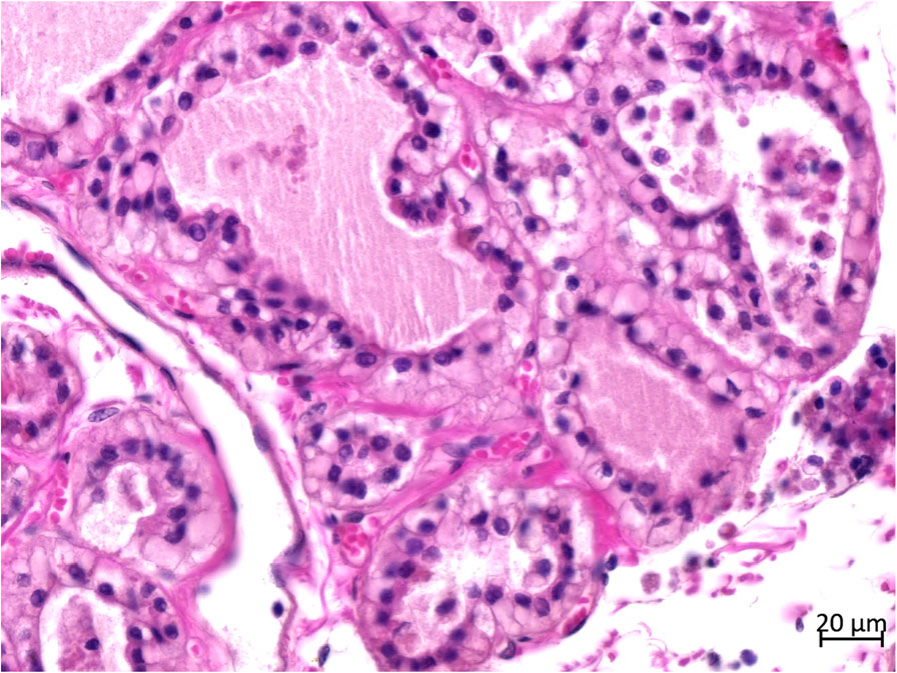
Histopathological features of the thyroid. A single or double layer of signet-ring-like HFCs is lined on the basement membrane of the thyroid follicles. A few signet-ring-like HFCs exfoliate the follicular lumen

Histopathologically, more than two-third of the thyroid tissue had lost its normal thyroid follicular structure. Abnormal follicles were irregular in size and shape in the hyperplastic goiter (Fig. 4). The follicles were lined with single or multiple layers of hyperplastic follicular cells (HFCs); in some follicles, these formed papillary projections into the lumen. Some of the regions in the follicular lumen in the local area were filled with exfoliated HFCs (Fig. 5). Most follicles were collapsed due to the lack of colloids. Some HFCs were cuboidal with eosinophilic cytoplasm, many red small granules, and large nuclei that were often situated in the center of the cell (Fig. 6). Other HFCs showed SRC differentiation, with nuclei pressed to one edge of the cell and distorted by cytoplasmic mucin that appeared as a single clear vacuole in HFCs and as a foamy, multivesicular cytoplasmic material in others (Figs. 7 and 8). There were no mitotic figures in the HFCs. A few normal follicles were also visible in the local areas of the thyroid tissue. Spleen atrophy with significantly reduced lymphocytes and numerous macrophages containing hemosiderin in the red pulp were obvious. Pulmonary congestion, mild fibroblast proliferation in the alveolar septa, large number of macrophages, and scattered neutrophils in some bronchioles were also observed. Obvious hemorrhagic foci of different sizes were observed in the brain, cerebellum, brain stem, and thymus. Moreover, large macrophages with necrotic cells were diffusely distributed in the thymus. Membranoproliferative glomerulonephritis with obvious proliferation of glomerular mesangial cells and multiple red-stained drops of protein in the glomerular cavity were observed. The tubular epithelial cells were slightly to moderately swollen, with lipofuscin in their cytoplasm. Necrosis in the upper half of the villi of the small intestine and hemorrhage in the submucosa of the colon were observed. However, there were no inflammatory cells in the tissues and organs.

**Fig. 9:**
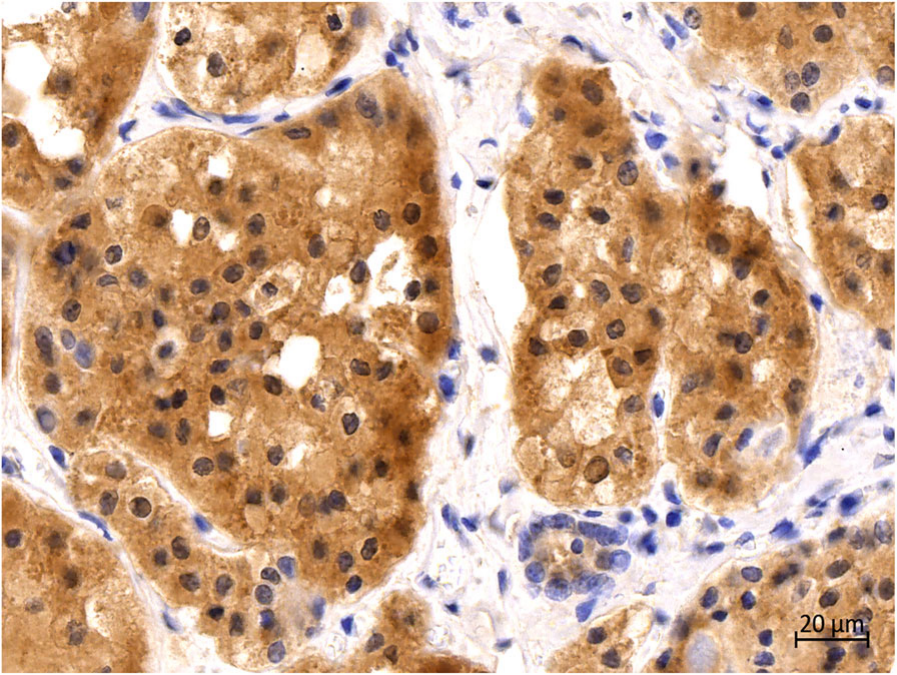
Immunohistochemical staining of the thyroid. TTF-1 was strongly positive in the cytoplasm of HFCs

**Fig. 10:**
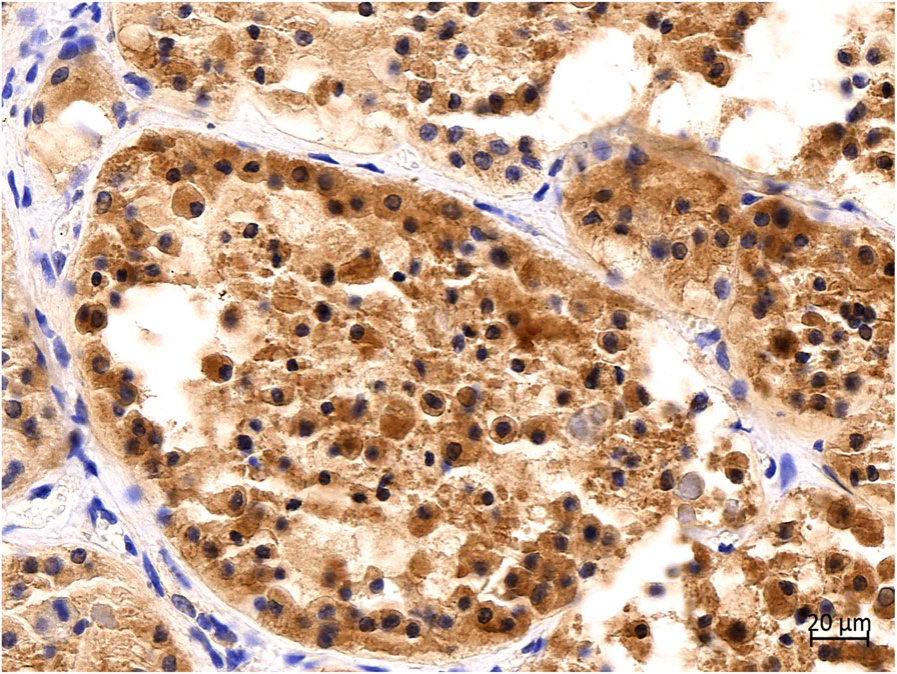
Immunohistochemical staining of thyroid. Thyroglobulin was strongly positive in the cytoplasm of HFCs

Immunohistochemical examination revealed positive reactions for monoclonal antibody TTF-1 (Fig. 9) and thyroglobulin (Fig. 10) in the cytoplasm in hyperplastic follicular epithelial cells of the thyroid tissue.

### Discussion and conclusions

Congenital goiter is mainly reported in humans [2, 7]. Although several animal cases have also been reported [2–8], it is a rare and underdiagnosed congenital endocrine disorder. The clinical signs of congenital goiter depend on the primary defect; different kinds of animals have different symptoms in different disease periods. The thyroid of an individual with congenital goiter is usually felt firm on palpation, and the cut surface is solid. The whole of the goitrous gland is uniformly involved without nodularity. Alveolar arrangement of epithelial cells is histologically similar to that of a solid adenoma [2]. The size and shape of the follicular lumina vary greatly, and there are irregular follicles devoid of apparent colloid [3]. The typical arrangement involves single rows of columnar vacuolated cells; the vacuoles often result in the columnar cells to become round, pushing the nucleus to the border of the cells and forming the appearance of a “signet ring” [2]. Many cells with distended rough endoplasmic reticulum vacuoles as well as with free ribosomes are also found [3]. Congenital goiter is often misdiagnosed as cancer [1]. In this case, no nodular hyperplasia was observed on the surface or section of the thyroid gland. More than two-third of the thyroid tissue had lost its normal thyroid follicular structure. Abnormal follicles were irregular in size and shape in the hyperplastic goiter. Many follicles were collapsed owing to the lack of colloids. Some HFCs were cuboidal with eosinophilic cytoplasm and many red small granules and large nuclei that are often situated in the center of the cell. There were no mitotic figures in HFCs. The main changes in the thyroid gland in this case are consistent with those reported in the literature.

It is noteworthy that some HFCs showed SRC differentiation in our case. Although it has been reported that follicular epithelial cells turn into SRC in congenital goiter [2], the appearance of SRC requires careful diagnosis. Previously, SRCs were mistakenly believed to be a unique feature of the adenocarcinoma of the stomach, breast, lung, and other organs, and the existence of SRC was indicative of a high degree of malignancy [14, 15]. In fact, SRCs can occur in both benign and malignant thyroid follicular tumors [18]. Although it is relatively rare for malignancies to metastasize to the thyroid gland, it is important to distinguish metastatic tumors from primary thyroid lesions [8]. In this case, thyroglobulin and TTF-1, which are the thyroid reliable origin markers [10, 15, 19, 20], were identified by immunohistochemistry in the HFCs, and there were no capsule, the follicular hyperplasia was diffuse, some HFCs showed SRC differentiation in combination with the low tumor atypia (as evidenced by the limited growth of tumor cells, lack of obvious mitosis and blood vessel invasion, and absence of tumor-like cells in other organs), resulting in the comprehensive diagnosis of congenital goiter with areas of SRC differentiation.

To the best of our knowledge, osseous cyst-like in both medial femoral condyles [21], embryonal rhabdomyosarcoma [22], subependymal glioneuronal hamartoma in the mesencephalic aqueduct [23], pelvic chondrosarcoma [24], skin papillomas [25], teratoma of the umbilical cord [26], and giant verrucous carcinoma [27] have been reported in giraffes. This is the first report of congenital goiter with areas of SRC differentiation in a giraffe.

The mechanism of SRC formation is uncertain, and few researchers have proposed theories to explain this mechanism. SRC morphology in thyroid follicular adenoma might result from the deposition of cytoplasmic material [15]. Therefore, SRCs may be the morphologic expression of an arrest in folliculogenesis [17], aborted TG exocytosis, TG degradation, dual differentiation of follicular cells, and other products formed owing to genetic changes. In this particular case, immunohistochemistry findings point toward TG accumulation in the cytoplasm as a vital a role in SRC formation. This is consistent with the reported results of several other studies [10, 11, 15, 18]. However, mucin enrichment, dysfunctional swollen endoplasmic reticulum or Golgi apparatus [9], and degenerate mitochondria [15] could also lead to the nuclei being pushed to the side of the cell. In our case, we could not definitively conclude whether these causes were associated with our results in the absence of further testing.

Our biochemical examination and blood test results showed that MCV, MCH, RDW, PLT, MONO, TBil, TIBC, HBDH, CREA, and other changes might be related to anemia, as supported by our histopathological findings. Changes in the levels of EO, LDH, A/G, CREA, CHE, PA, Zn, and EOSR were related to the giraffe’s poor appetite, poor body condition, wasting, malnutrition, exhaustion, and multiple organ damage, whereas changes in the levels of CK-MB, HDL-C, and HBDH were related to myocardial injury. Changes in the levels of AMY, UREA, and CO_2_CP were related to kidney damage and changes in the levels of ALT, PA, and TBA were related to liver damage. Damage to the kidneys and liver were confirmed via microscopic examination of the tissue. The changes in the levels of UREA, ALT, and GLU were consistent with the literature on congenital goiter, and the changes in the level of Ca were contrary [7]. The decrease in the level of Ca may be related to necrotizing enteritis, leading to decreased Ca absorption. In the absence of a palpable lump in the neck, thyroid lesions were not initially considered; therefore, no related tests were performed for assessing thyroid function. Therefore, the changes in the levels of thyroid hormones, such as thyroxine and triiodothyroxine, remain unclear, making it impossible to evaluate thyroid and pituitary functions. A postmortem pathological examination combined with immunohistochemistry staining revealed that the giraffe did in fact have this rare type of thyroid lesion. This juvenile giraffe may have been born to an iodine-deficient mother, in which case, the animal would have had goiter, and the thyroid glands would have been hyperplastic. After development, SRC differentiation region appeared. Iodine deficiency and development led to hypothyroidism, decreased synthesis and secretion of thyroid hormones, subsequent secretion of excess TSH from the pituitary gland, metabolic disorders, stunted growth and development, and inhibition of the central nervous system, eventually leading to complete system failure and death. Therefore, veterinary pathologists should consider congenital goiter during the differential diagnosis of thyroid lesions.

In conclusion, this study comprehensively diagnosed congenital goiter with areas of SRC differentiation in a juvenile giraffe based on clinical symptoms, autopsy, histopathology, and immunohistochemistry. To the best of our knowledge, this is the first reported case of congenital goiter with areas of SRC differentiation in a giraffe.

## Data Availability

The data generated during the current study are included in this case report.

## Abbreviations

SRC: Signet ring cell
HFC: Hyperplastic follicular cell
TSH: Thyroid-stimulating hormone
TTF-1: Thyroid transcription factor-1

## References

[1] Tonghua Liu. Endocrine System. Tonghua Liu’ Diagnostic Pathology, 4 th ed. Beijing: People’s Medical Publishing House; 2018. pp. 375–412. (in Chinese).

[2] Rac R, Hill GN, Pain RW, Wulhearn CJ (1968). Congenital goitre in Merino sheep due to an inherited defect in the biosynthesis of thyroid hormone. Res Vet Sci.

[3] Pammenter M, Albrecht C, Liebenberg W, van Jaarsveld P (1978). Afrikander cattle congenital goiter: characteristics of its morphology and iodoprotein pattern. Endocrinology.

[4] Homerosky ER, Johnsen M, Steinmann M, Matejka C, Jelinski MJ (2019). An outbreak of congenital goiter and chondrodystrophy among calves born to spring-calving beef cows. Can Vet J.

[5] Medeiros-neto G, Targovnik HM, Vassart G (1993). Defective Thyroglobulin Synthesis and Secretion Causing Goiter and Hypothyroidism. Endocr Rev.

[6] Rijnberk A, De Vijlder JJ, Van Dijk JE, Jorna TJ, Tegelaers WH (1977). Clinical aspects of iodine metabolism in goats with congenital goitre and hypothyroidism. Br Vet J.

[7] Bojanić K, Acke E, Jones BR (2011). Congenital hypothyroidism of dogs and cats: A review. N Z Vet J.

[8] Dodgson SE, Day R, Fyfe JC (2012). Congenital Hypothyroidism with Goiter in Tenterfield Terriers. J Vet Intern Med.

[9] Yalta T, Elagoz S, Uyar M, Topuz O, Ozer H, Tuncer E (2010). Signet ring cell adenoma of the thyroid: a very rare entity. Med Princ Pract.

[10] Sassi SH, Tangour M, Mrad K, Abbes I, Amor HB, Romdhane KB (2010). Signet-ring cell follicular adenoma of the thyroid. APMIS.

[11] Farhat NA, Onenerk AM, Krane JF, Dias-Santagata D, Sadow PM, Faquin WC (2017). Primary Benign and Malignant Thyroid Neoplasms with Signet Ring Cells: Cytologic, Histologic, and Molecular Features. Am J Clin Pathol.

[12] Zhou L, Li W, Cai S, Yang C, Liu Y, Lin Z (2019). Large tumor size is a poor prognostic factor of gastric cancer with signet ring cell: Results from the surveillance, epidemiology, and end results database. Med (Baltim).

[13] Chetty R (2011). Thyroid Follicular Adenoma Composed of Lipid-Rich Cells. Endocr Pathol.

[14] Pagni F, Ronchi S, Di Bella C, Serra G, Costantini M, Leone BE (2012). Signet-ring cell differentiation in FNA cytology of a primitive thyroid carcinoma. Cytopathology.

[15] Romero-Rojas AE, Diaz-Perez JA, Mastrodimos M, Chinchilla SI (2013). Follicular Thyroid Carcinoma with Signet Ring Cell Morphology: Fine-Needle Aspiration Cytology, Histopathology, and Immunohistochemistry. Endocr Pathol.

[16] Wheeler YY, Stoll LM, Sheth S, Li QK (2010). Metastatic signet ring cell carcinoma presenting as a thyroid nodule: Report of a case with fine-needle aspiration cytology. Diagn Cytopathol.

[17] Fellegara G, Rosai J (2007). Signet ring cells in a poorly differentiated Hurthle cell carcinoma of the thyroid combined with two papillary microcarcinomas. Int J Surg Pathol.

[18] Chiofalo MG, Losito NS, Fulciniti F, Setola SV, Tommaselli A, Marone U (2012). Axillary node metastasis from differentiated thyroid carcinoma with hürthle and signet ring cell differentiation. A case of disseminated thyroid cancer with peculiar histologic findings. BMC Cancer.

[19] Wang J, Guli QR, Ming XC, Zhou HT, Cui YJ, Jiang YF (2018). Primary mucinous carcinoma of thyroid gland with prominent signet-ring-cell differentiation: a case report and review of the literature. Onco Targets Ther.

[20] Ricardo VL, Darya B, Elham K (2011). Papillary Thyroid Carcinoma Variants. Head Neck Pathol.

[21] Basu C, Stoll AL, Dixon J, Molenaar FM, Flach E, Smith KC (2016). Osteochondrosis in the distal femurs of an adult reticulated giraffe (giraffa camelopardalis reticulata): macroscopic, radiologic, and histologic findings. J Zoo Wildl Med.

[22] Woc-Colburn M, Murray S, Boedeker N, Viner T, L Fleetwood M, Barthel C. T, et al. Embryonal rhabdomyosarcoma in a Rothschild’s giraffe (Giraffa camelopardalis rothschildi). J Zoo Wildl Med. 2010;41(4):717–20. 10.1638/2009-0195.1 21370656

[23] Koehler J, Cox N, Passler T, Wolfe D (2012). Subependymal glioneuronal hamartoma in the mesencephalic aqueduct of a giraffe. J Zoo Wildl Med.

[24] Juan-Sallés C, Martínez G, Garner MM, Parás A (2008). Fatal dystocia in a giraffe due to a pelvic chondrosarcoma. Vet Rec.

[25] Karstad L, Kaminjolo JS (1978). Skin papillomas in an impala (Aepyceros melampus) and a giraffe (Giraffa camelopardalis). J Zoo Wildl Med.

[26] Murai A, Yanai T, Kato M, Yonemaru K, Sakai H, Masegi T (2007). Teratoma of the Umbilical Cord in a Giraffe (Giraffa camelopardalis reticulata). Vet Pathol.

[27] Burgdorf WH, Sullivan MS, Jensen J, Jordon FB (1984). Giant verrucous carcinoma in a giraffe. Am J Dermatopathol.

